# Supplementary material for: SARS-CoV-2 Variants from Long-Term, Persistently Infected Immunocompromised Patients Have Altered Syncytia Formation, Temperature-Dependent Replication, and Serum Neutralizing Antibody Escape
Source: Viruses. 2024 Sep 9;16(9):1436. doi: 10.3390/v16091436 (PMC11437501; doi:10.3390/v16091436)
Supplement: Supplementary file 1 [file viruses-16-01436-s001.zip › 240913 CHLA manuscrippt supplementary figures updated.pptx]

## Slide 1
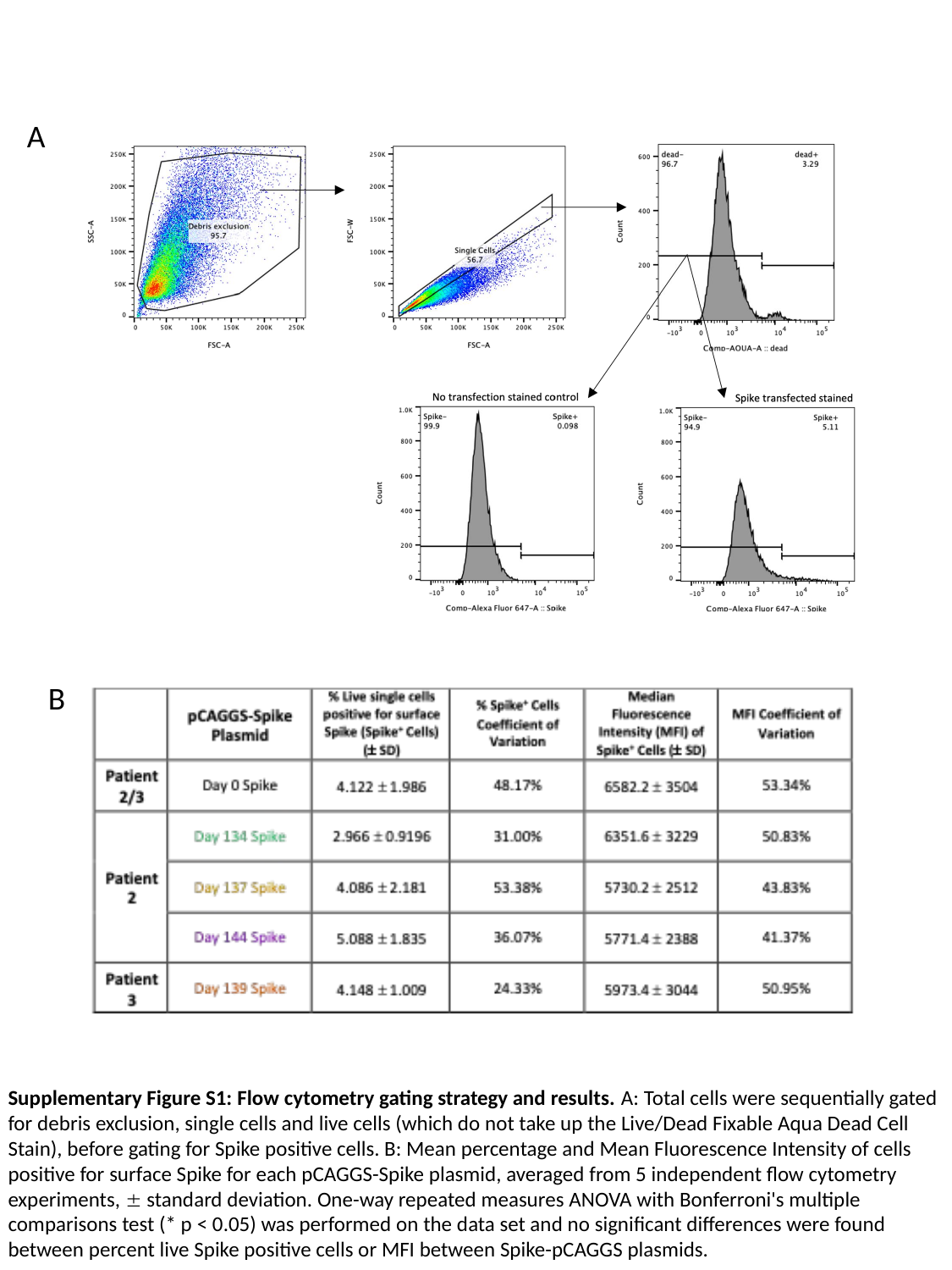

A
B
Supplementary Figure S1: Flow cytometry gating strategy and results. A: Total cells were sequentially gated for debris exclusion, single cells and live cells (which do not take up the Live/Dead Fixable Aqua Dead Cell Stain), before gating for Spike positive cells. B: Mean percentage and Mean Fluorescence Intensity of cells positive for surface Spike for each pCAGGS-Spike plasmid, averaged from 5 independent flow cytometry experiments, ± standard deviation. One-way repeated measures ANOVA with Bonferroni's multiple comparisons test (* p < 0.05) was performed on the data set and no significant differences were found between percent live Spike positive cells or MFI between Spike-pCAGGS plasmids.

## Slide 2
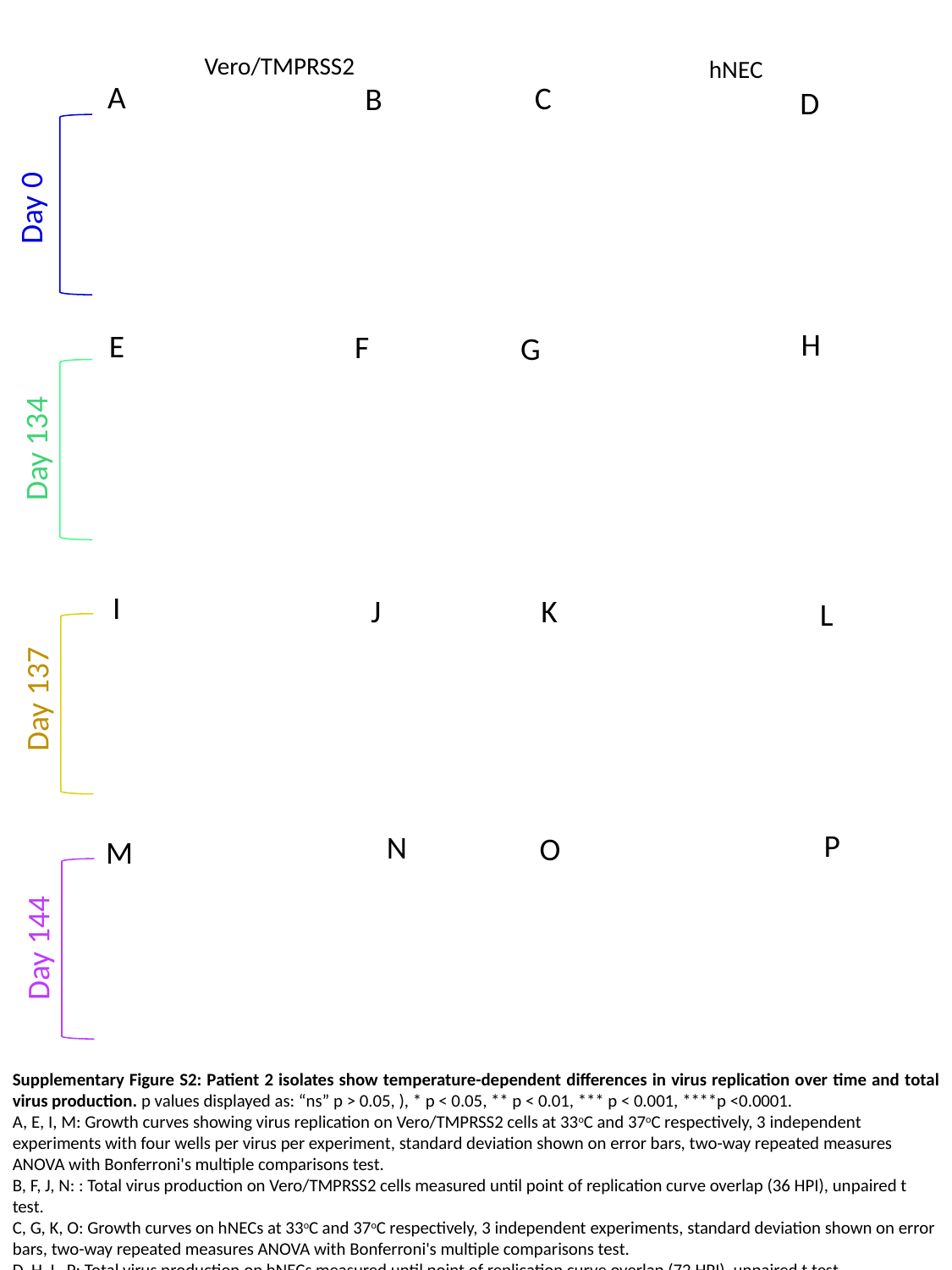

Vero/TMPRSS2
hNEC
A
C
B
D
Day 0
H
E
F
G
Day 134
I
J
K
L
Day 137
P
N
O
M
Day 144
Supplementary Figure S2: Patient 2 isolates show temperature-dependent differences in virus replication over time and total virus production. p values displayed as: “ns” p > 0.05, ), * p < 0.05, ** p < 0.01, *** p < 0.001, ****p <0.0001.
A, E, I, M: Growth curves showing virus replication on Vero/TMPRSS2 cells at 33oC and 37oC respectively, 3 independent experiments with four wells per virus per experiment, standard deviation shown on error bars, two-way repeated measures ANOVA with Bonferroni's multiple comparisons test.
B, F, J, N: : Total virus production on Vero/TMPRSS2 cells measured until point of replication curve overlap (36 HPI), unpaired t test.
C, G, K, O: Growth curves on hNECs at 33oC and 37oC respectively, 3 independent experiments, standard deviation shown on error bars, two-way repeated measures ANOVA with Bonferroni's multiple comparisons test.
D, H, L, P: Total virus production on hNECs measured until point of replication curve overlap (72 HPI), unpaired t test.

## Slide 3
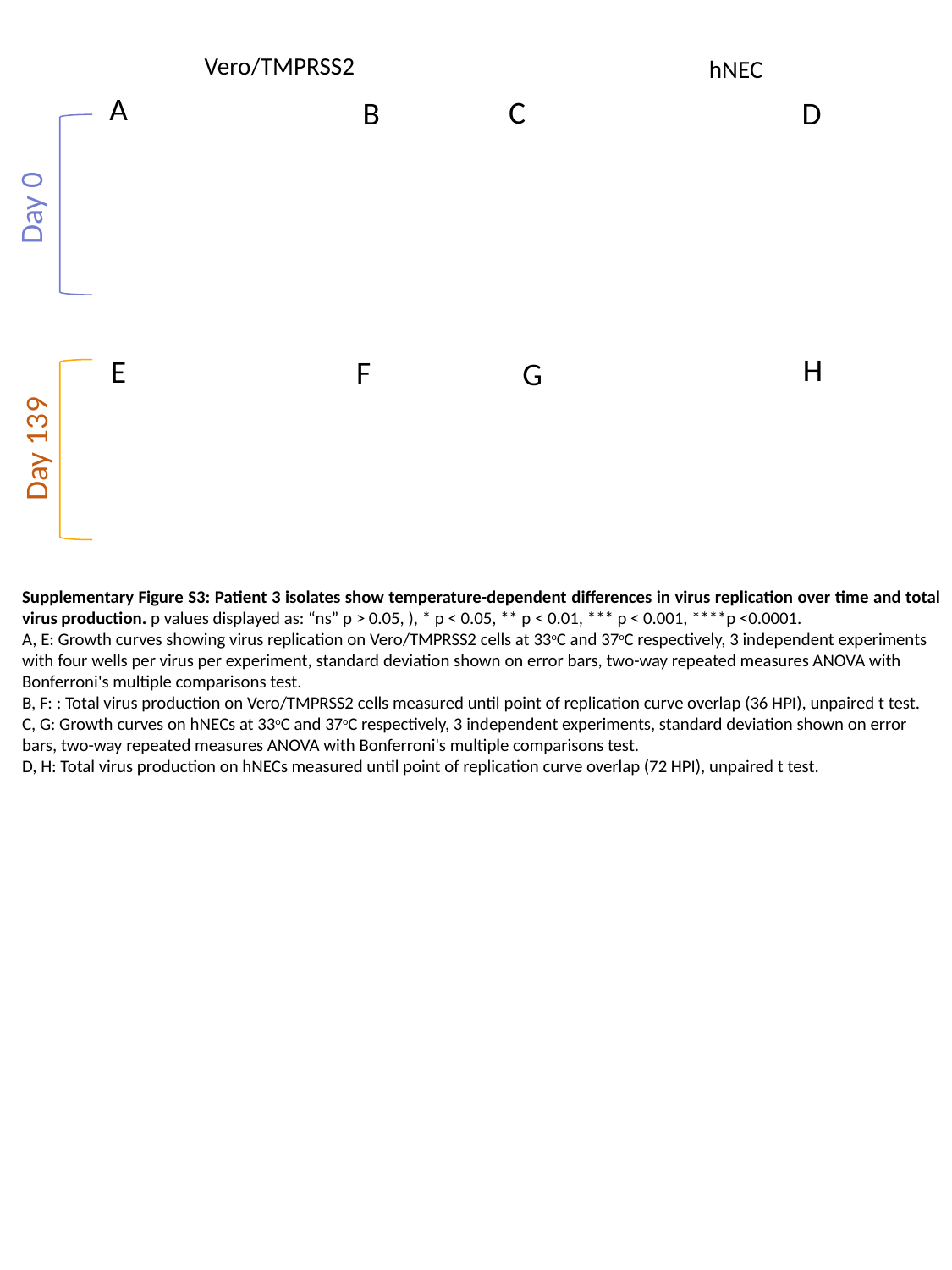

Vero/TMPRSS2
hNEC
A
C
B
D
Day 0
H
E
F
G
Day 139
Supplementary Figure S3: Patient 3 isolates show temperature-dependent differences in virus replication over time and total virus production. p values displayed as: “ns” p > 0.05, ), * p < 0.05, ** p < 0.01, *** p < 0.001, ****p <0.0001.
A, E: Growth curves showing virus replication on Vero/TMPRSS2 cells at 33oC and 37oC respectively, 3 independent experiments with four wells per virus per experiment, standard deviation shown on error bars, two-way repeated measures ANOVA with Bonferroni's multiple comparisons test.
B, F: : Total virus production on Vero/TMPRSS2 cells measured until point of replication curve overlap (36 HPI), unpaired t test.
C, G: Growth curves on hNECs at 33oC and 37oC respectively, 3 independent experiments, standard deviation shown on error bars, two-way repeated measures ANOVA with Bonferroni's multiple comparisons test.
D, H: Total virus production on hNECs measured until point of replication curve overlap (72 HPI), unpaired t test.
