# Supplementary material for: SARS-CoV-2 Variants from Long-Term, Persistently Infected Immunocompromised Patients Have Altered Syncytia Formation, Temperature-Dependent Replication, and Serum Neutralizing Antibody Escape
Source: Viruses. 2024 Sep 9;16(9):1436. doi: 10.3390/v16091436 (PMC11437501; doi:10.3390/v16091436)
Supplement: Supplementary file 1 [file viruses-16-01436-s001.zip › Supplementary Table S1.pdf]

Oligo Name	Sequence (5' to 3')	nt	Description
F1	CTCTTCTTAGTAAAGGTAGAC	21	CoV-2_S PCR and sequencing
R2	CTGAAGGAGTAGCATCCTTG	20	CoV-2_S PCR and sequencing
F3	TATTCTAAGCACACGC	16	CoV-2_S sequencing
F4	GATTTTACAGGCTGCG	16	CoV-2_S_RBD PCR and sequencing
F5	CAAACACGTGCAGGCTG	17	CoV-2_S sequencing
F6	GCTCAATACACTTCTGC	17	CoV-2_S sequencing
R7	TCTACTGATGTCTTGGTC	18	CoV-2_S_RBD PCR and sequencing

Supplementary Table S1: overlapping forward and reverse primers for Spike Sanger sequencing.
